# International Animal Protection Society Leadership: The Right People for the Right Issues

**DOI:** 10.3390/ani8060089

**Published:** 2018-06-07

**Authors:** Michelle Sinclair, Clive J. C. Phillips

**Affiliations:** Centre for Animal Welfare and Ethics, School of Veterinary Science, University of Queensland, Gatton 4343, Queensland, Australia; c.phillips@uq.edu.au

**Keywords:** animal welfare, leadership, international, animal protection, strategy, organization, human resources, succession planning, non government organization, NGO

## Abstract

As the increasing body of scientific information about the experiences of other species and their ability to suffer becomes available to those working within the field of animal welfare, the amount of potential issues to address also increases. Carefully choosing issues to address, and indeed leaders to drive the cause forward, has the potential to significantly increase the efficacy of the international animal welfare movement. Within this study 15 leaders of major international animal welfare organizations were interviewed about their experiences, thoughts and strategies, which have been primarily acquired through long-term exposure to the movement, and endeavors of trial and error. After thematic analysis, key themes are presented, along with strategies and cautions that may be beneficial to the animal welfare movement. Animal welfare leaders suggested a focus on issues that fitted well with their organizations’ remit and were not too broad, to avoid spreading resources and expertise too thin. A utilitarian framework was also considered important, aiming to improve the lives of as many animals as possible for the resources deployed. Good leaders were believed to have passion for their cause, not just for animals, and an ability to build and lead good teams, hence good interpersonal human skills were also perceived as essential. It is concluded that establishing what makes a good animal welfare leader could offer useful direction for future engagement of successful leaders in this field.

## 1. Introduction

As a modern movement and field of science, Animal Welfare has exponentially grown over recent decades, to one that has now achieved global recognition. Measured in terms of social media attention (public support) rather than the traditional terms of net worth (donor generosity), animal-based charitable causes now occupy three of the top 20 globally ranked positions for supporters, including People for the Ethical Treatment of Animals (PETA), which has 5.4 million supporters on Facebook [1,2,3]. US domestic animal welfare charity Humane Society US has 2.6 million supporters on Facebook, where Amnesty International Global has only 2 million, Save the Children 1.8 million, and World Vision Global 92 thousand [3]. Despite not making the top 10 richest causes, reserved for long established social movements managing poverty, housing, healthcare, education and emergency relief aid for humans [4], these statistics show a growing movement support base of significant proportions. 

The animal welfare movement has advanced significantly in western countries in the last 30 years [5]. The work of Rene Descartes (popularized in the nineteenth century) that maintained that non-human animals were nothing more than machines, to which pain is insignificant, is now mostly a figment of past scientific misjudgments [6]. The birth of animal welfare science, and the simultaneous growing awareness and scientific understanding of animal suffering has seen a focus placed on a growing number of species. First, the factory farming of vertebrates [7], more recently, the sentience of cetaceans for example [8], and now, after proving fish have the ability to feel pain and to suffer, animal welfare science has a major focus on fish welfare, with widespread implications [9]. It seems, the more scientific knowledge develops, the more animal welfare issues exist for the movement to focus on. With so many potential issues, and so much work to do to bring animal welfare standards in line with public expectations in some regions, how do leaders in the animal welfare advocacy movement choose which issues to focus on? This study aims to investigate this question, by interviewing current international animal welfare leaders to identify factors that drive their decisions.

*“The pessimist complains about the wind. The optimist expects it to change. The leader adjusts the sails.”*

John Maxwell

As with any organization, those within the social movement organizations are only as good as the people working and living within them, so the vision and ability of the people leading them becomes paramount to success. In fact, leading within a non-profit environment brings with it additional challenges. “The role of leadership in social movements goes well beyond that of the stereotypical charismatic public persona with whom they are often identified” [10]. This is due to the nature of social organizations as “voluntary, decentralized, and self-governing; they are volatile, dynamic, and interactive; participants are motivated by moral claims, but results depend on strategic creativity; and their capacity to make things happen depends on their ability to mobilize broad levels of commitment” [10]. 

Leading an animal welfare organization brings with it further challenges, both external and internal, by its very purpose: advocating for other species. There is little doubt that it requires a variety of competencies and mix of traits in order to be most successful. For this purpose, effective human resources practices—recruiting and succession planning to ensure the right people are in the right place at the right time—underpins the success of non-profit initiatives. Recruiting the best possible leaders into these roles could be assisted by knowledge from current successful leaders. 

In addition to identifying animal welfare issues to pursue, this study aims to investigate effective human resources practices by interviewing international animal welfare leaders about their experiences and their opinions on effective leadership in the movement. This knowledge could assist in the early identification of potential future leaders, in the development plans of those leaders, and ultimately aid in the success of the organizations that they lead. 

The overall aim of this research is to assist in capacity building the international animal welfare movement to enable it to be more people focused. Just as people are the ultimate target of all animal welfare campaigns, they are also leading them.

## 2. Methodology

Ethical approval for the study was provided by the University of Queensland Human Ethics Committee (approval number 2017000628). Relevant organizations were considered to be the major international animal welfare charities on the basis of three aspects of their activities, international, large scale, and a high level of brand recognition. In total leaders of 13 major international animal welfare organizations were approached, a total of 15 leaders from 10 organizations accepted, 1 declined, and 2 did not reply to the invitation. The reason for declining the invitation by 1 organization was a perceived lack of knowledge to comment on the subject. Participating organizations were Animals Asia, Humane Society International, Compassion in World Farming, International Animal Rescue, International Fund for Animal Welfare, World Animal Protection, Royal Society for the Protection against Cruelty to Animals UK, Vets Beyond Borders, The Donkey Sanctuary and People for the Protection of Animals.

The leaders of the organizations were chosen by the lead researcher (MS) and the organizations themselves, based on their assessment of individual’s role within the organization, which required them to be a CEO, operations managers or high-level coordinators working in an international capacity. These leaders were approached via email and requested to take part in a 30 min semi-structured interview via Skype to talk about their experiences regarding successful and unsuccessful projects in animal welfare. Prior to the interview, the leaders were given an information sheet to review, outlining the confidentiality of the interview, its voluntary nature, their right to withdraw at any time, and details regarding the topic and interview logistics, such as time requirement and platform. Upon request, all leaders also gave verbal permission at conclusion of the interview to use the name of their organization as participating within the study. Fifteen leaders accepted this invitation to participate and a time was booked for interview.

In the interview, leaders were asked:

- How long they have been in animal welfare.
- What drew them to animal welfare.
- What makes a good international animal welfare leader.
- To describe the most successful international animal welfare projects they have been involved in.
- To describe the least successful international animal welfare projects they have been involved in.
- What made those projects successful/unsuccessful.

Responses from all interviewees to those questions marked in italics are presented in this paper, with the remaining to be presented in a later paper to allow complete discussion and logical division of data. All interviews were recorded with a voice recorder after verbal permission from each leader at the onset of the interview, and complete transcripts were subsequently prepared verbatim. This study was approached in a familiar way to sociological research across fields (including that of other social progress initiatives such as environmental conservation), with the purpose of identifying and understanding evidence-based approaches as solutions for advancing the movement [11,12,13].

### Data Analysis

Thematic analysis was aided by appropriate software [14] and through manual inspection of source data, by the same researcher that conducted the interviews (MS). Coding themes were identified using text frequency and word search functions in NVivo, in addition to manual familiarization with the data. Words were chosen for analysis based on the amount of times they appeared overall; however, joining words (such as ”and”) were excluded, along with words that had no relevance or usefulness to the node/theme, or to the study. By extracting the main themes that emerged from the analysis, including key pieces of connective information, mind maps were created to easily visualize the themes and their relationship with each other. Data within each node (identified reoccurring theme) was then further analyzed for detail, including frequency of each theme.

## 3. Results

### 3.1. Choice of Animal Welfare Issues to Pursue

#### 3.1.1. Fit to Organizational Mandate, and Focus

The most common response was that primarily an issue must fit the specific mandate established by the institutional strategy, often by boards and committees (Table 1).

Five leaders specifically mentioned the need to “keep it very tight within those program areas”, and that potential animal welfare activities need to fulfil this mandate. A few also suggested that this mandate was best if it was specific, as “it is better to do a few things really well, than everything really badly”. This related to the theme of “targeted focus”, where comments such as “we need to stay focused on specific issues, otherwise we just get stretched too thin”, were emphasized by three of the 15 leaders. Two leaders stated that the potential activities are also evaluated on their ability to enhance other programs they are currently running, and how it will strengthen the global program. Five of the 15 leaders referred to the clarity of their mandate, making the decision to be involved in a potential animal welfare activity very simple. One leader referred to the overwhelming amount of potential animal welfare issues to pursue and stated that this was more reason to stay focused on one issue only. One leader stated that, “It’s easy to dilute when you venture out into new topics, but that doesn’t really give justice to your core programs. Focus on the core issues with which you start, and don‘t divert from it too much.”

#### 3.1.2. Best for the Most and Scope of Suffering

The second most common theme identified in choosing issues to pursue was utilitarian; selecting the issues that will affect the most number of animals followed by the scope of suffering (duration and intensity). A third of leaders interviewed made comments such as, “I think it’s easy when you think what affects the greatest number of animals”, “it’s best for the most”, and “we only pick issues with the maximum animal suffering, and there is of course a maximum reduction of suffering for dollars spent, and hence battery cages, animal testing, wildlife trade, puppy mills, cruelty response—these are the areas we pick”. In addition to the amount of animals suffering, the same leaders also stated that they also assess the length of time an animal suffers, and how intense the suffering is. A few leaders state their choice to focus on battery cages for hens because, “it is lifelong, and is complete immobilization”, likewise another, regarding the farming of bears for bile. While two thirds of leaders did not cite pragmatic or utilitarian reasons for choosing their animal welfare issues to pursue, three others did mention their choices based on the wider notion of ”impact”; however, that was not further defined. 

#### 3.1.3. Opportunity for Collaboration or Leverage

Four of the 15 leaders made mention of specific opportunities that led them to choose certain activities on their portfolio (Table 1). These opportunities mostly centered around opportunity to collaborate with a government, or a key industry or commerce. Half of these leaders discussed their vigilance for opportunities to tie their causes to other issues of social concern, such as climate change, gender empowerment, and food security, which in turn creates wider possibilities and more opportunities for impact and sustainability. 

#### 3.1.4. Ability to Contribute

Four of the 15 leaders noted that their ability to bring something meaningful and useful to the issue was an important criterion when deciding if to pursue the issue (Table 1). In one instance this also included consideration of who else was working in the area, and if they would have the ability to bring something new. One leader stated, “Do we really have an opportunity for change here or are we going to bang our head against the wall?”, while another summed up the sentiments by stating, “There are some areas that have severe animal welfare problems, but our ability to change it is minimal, and therefore if we have minimal resources, limited resources rather, then we ought to focus on problems that we can fix, at least at this stage. Doesn’t mean you don’t try to address some of those issues long term so you can get it to a stage when you eventually could tackle them, but in the end that’s the key thing.”

### 3.2. International Animal Welfare Leadership

#### 3.2.1. Attraction to Animal Welfare Leadership

Fourteen leaders answered this question out of 15 and all but three of these were passionate about animal welfare. Most stated an affinity with non-human species, and others that they felt strongly about the cause of animal welfare, and uncomfortable with the injustice of poor animal treatment. Six stated that they had witnessed something that affected them, five stated they felt they could add something to the cause, and one had an academic history in animal welfare. The remaining two leaders were recruited into the cause on the basis of specific skills they brought to the table.

#### 3.2.2. Time in the Industry

Nearly all leaders in this study (13 of 15) had been within the animal welfare industry long term, for over 10 years. Five of these leaders had been leading the same organization they were integral in founding and claim a minimum of 20 years working in animal welfare. Four of the leaders were volunteering or engaged with animal welfare causes from a young age, and a few stated that it was an industry that they would be dedicated to into the future.

### 3.3. International Animal Welfare Leadership Traits

#### 3.3.1. Passion

Passion was the most commonly referred to trait, with nearly two thirds of respondents raising it as important (Table 2). “I think having leaders who are hardworking and undoubtedly passionate to the cause is really the most important thing.” Another leader stated “You’ve got to have passion. You have to want to make this happen, there are so many reasons why it can’t.”

Some leaders cautioned that while passion is of paramount importance, it is dangerous when unaccompanied by focus. “They’re so passionate and they want to take on every issue in the farm animal movement; that’s not being effective.” Another leader directly stated that passion can also bring about demise, when not coupled with the right ‘sense for it’. “I’ve seen a lot of passionate people burn out, very soon, very very fast; because we see so much suffering on our field trips on a daily basis you know.”

Another cautioned that the passion of a good leader should not only be for animal welfare causes, but also for people. With enthusiasm, “you should be able to inspire people around you”, which is hard to do without a passion for people and potential team achievements. 

#### 3.3.2. Understand the Issue and Society

Approximately half of the leaders interviewed stated a need to “understand” the issue they are leading, and the environment and culture in which they are leading it (Table 2). “I think maybe I have repeated it many times, (but they need to) understand the society; understand the policy—the local policy; and understand the country. It is very important.” Another stated that a great leader will, “understand the great variances there are in cultural differences, because that’s huge”, while another stated, “you need to know the region that you’re working in really well, that’s the first thing”. 

By “understanding”, another leader elaborated that a great leader “need(s) to really understand the local custom or culture, and in equal part, that person needs to understand the perception of the ordinary people on animal welfare issues, or even on animal issues”.

One leader made a point of stating that a good leader does not just understand their animal welfare issues and the society, but also where the issue fits in a broader context of wider issues. “I think it’s the ability to drive change through understanding the context that makes a more successful animal welfare leader.”

After stating that a leader needs to become a regional specialist, one leader added a caveat to caution leaders against overstating their understanding levels and overextending beyond their ability. Another leader stated the ability of a good leader to identify and hire good teams with the required knowledge and understanding to support them.

#### 3.3.3. Ability to Build a Capable and Engaged Team

“Obviously a good animal welfare leader does not need to be a technical expert, but needs technical expertise behind them, I guess, to have that in terms of the organization.” “You’ve got to have really good people in every position that are able to take up the challenge”… “I think you need to have an all-encompassing view, but to enable people to do things … I have no problem delegating, I have too much to do anyway”. 

Three leaders stated the importance of empowering their team and leading from behind (Table 2).

“Rather than, kind of, being the person sort of spearheading it publicly, actually passing on my knowledge and skills to the people that I work with and kind of building them up to a point where they can actually do the actual one on one negotiations”. Another leader stated “that is down to excessive non-stop communication with every member of your team really… it is exhausting; it is so easy to want to concentrate on the program, the animals and the issue—but you need to focus on the people leading those areas… ultimately it is communication and empathy for your staff”.

Delving deeper into the data, it emerged that a key to building an engaged and capable team is the ability to relinquish control of key strategic aspects of the business, and to be able to relinquish personal credit or accolades following successes with campaigns.

“I think a (good) leader (is someone) who doesn’t control branding, who lets the local staff or the volunteers take the best decision and one who guides.” “I think that animal welfare today has become ‘eminence-based’ animal welfare rather than evidence-based animal welfare … the people themselves become larger than life, and I feel that a good leader has to put himself or herself behind, and change the movement from an eminence-based movement to an evidence-based movement … I feel like whoever understands and does that is a leader who should lead in animal welfare movements.” “Animals don’t have time for one charismatic person.”

#### 3.3.4. Bigger Picture Ambition

Another theme trait of good animal welfare leaders is bigger picture vision, and with that, ambition. “I’ve seen too many models where people have either been willing to plod on and not change the world, and frankly as far as I’m concerned if we were not in it to change the world we shouldn’t be here.”

This includes a purposeful contentment in being behind the scene. “Even if I didn’t touch an animal for the next 10 years and I only talked to 100 people a day and asked them to be kind to animals, I’d bring about more change than going to animals directly.” Another leader stated “look at the root cause of the problem, you may run a shelter and spend all your money and all your life taking care of 40 dogs, unless you stop the overpopulation of the dogs on the streets and where they’re coming from, you waste your time and your money and your resources … so these are the things that I think make a very good animal welfare leader”.

#### 3.3.5. Collaborative

The ability to be collaborative, both internally within the organization, and externally to the organization was also identified as an important trait of a successful animal welfare leader. One leader stated “being collaborative and being able to make those connections with other organizations, other stakeholders outside, and also to bring their own organization along”, were key hallmarks of a successful leader, while three leaders highlighted the importance of also being able to network and collaborate with other charities, and “not being insular, working with other charities, opening up”. Another stated the importance of the collaborative abilities of a good animal welfare leader by stating they “have to deal with ministers and diplomats, bureaucrats, their community, the groups that hate animals, the groups that love animals, (and) get them all at a table, talk some sense to them, listen to them, and find strategies, (and) make them feel committed … so I think that’s the number one take away for me”. This includes the ability to collaborate with the stakeholders they’re trying to change. Another stated the required ability to “work towards … finding something where we have some common ground and then engaging that person … (to) that point where we can actually sit down with (them) and have that dialogue, and then come up with that compromise”. One leader explicitly stated “a good animal welfare leader needs to be a people person. I hear a lot of people say, ‘I love animals, but I hate humans’, and I think that’s something we can do away with … we don’t need those people in front of people, we need a people-loving person, because the root cause of all animal suffering is human behavior”. For this leader, this begins by recruiting “people-people”. “If I have to hire someone for a country, I take them out for a beer with a large group of people and I see how they interact with them.”

#### 3.3.6. Flexibility, Reason and Moderation

Through collaboration, it may sometimes be necessary to arrive at flexible outcomes and compromises, and as such, flexibility appeared as another theme associated with good leaders in international animal welfare. “Accepting that … we might not be able to get everything that we want but … gradually we might be able to actually … get at least a working relationship with that person and see how things go.” Within the data, flexibility was most commonly associated with cultural and societal differences. Where it was raised there seemed consensus that, where operating internationally, this trait was required of a good leader. “(They) need to be able to be flexible and also accommodating of differences in people, in culture and society”, “they need to be someone who is flexible, someone who can walk into a situation in a country and listen, and then assess the situation, but understand the great variances there are in cultural differences … because that’s huge … you have to be able to address that in a commonsense way so that you can give a grounded reaction and a grounded assessment of what’s going on”. The importance of listening was sometimes used in the context of understanding, tied to flexibility, reason and moderation. A good animal welfare leader is “someone who is flexible, who … can listen, rather than going in and saying it’s got to be done this way’ because it’ll never work … and someone who is prepared to think about it and make a rational decision that is best for the animals”. Lastly, one leader responded directly to the themes of reason and moderation, more so, a leader’s ability to use reason and moderation by referring to science. “Within our means, I think we can do a good and much better job of letting science play its part in animal welfare, and accepting that, rather than fighting that.”

#### 3.3.7. Focus, Drive, Persistence and Determination

These themes were pooled together here, as they are contextually very similar throughout the data, and were used interchangeably. When asked what constitutes a good leader in animal welfare, one leader stated “I think we just crack on a bit, we’re driven. You do have to be driven … and driven by outcome rather than personally. I don’t think you achieve much if you’re ego driven.” Another stated “I would say the people who are most effective are people who can just focus, say ‘ok, this is what we’re doing’, people who are focused in that way.” Another stated “very driven, hungry for success, willing to do what it takes … within reason … within reason is in concert with the organization identity”. The focus and drive mostly referred to a focus on an achievable strategy and outcome, rather than on the issue they were addressing. A good leader is someone “who has a very clear aim and strategy for achieving that is realistic”. Another stated that an individual can be a great leader and make a great impact, despite limited resources available to animal welfare organizations, “by being very outcome focused, and being able to marshal all the resources that an animal welfare organization can bring, you can have that impact and inspire people to believe that … I think if you do that, you can have a large impact. It’s often a misconception among the general populace that animal welfare groups are huge and we get loads of money, compared to the big NGOs let alone the big agencies, we’re tiny”. Another leader addressed the theme of focusing on tangible outcomes as something that is commonly missing from leaders of animal welfare organization, to the detriment of their cause, “I think often a common flaw with the over-ambition, comes a lack of focus on outcome and I see it more often in campaigning organizations. There’s a lot of cred’ing (credit seeking); ‘we got x number of newspaper headlines, we’ve done this we’ve done that’, but actually if you look at the issue things haven’t changed. They might be higher profile, but animals are still suffering”. Lastly, one leader states that passion, backed by focus and determination, is the key tenet of a good animal welfare leader, and that in discussing determination, the word “no” drives them. “What do I mean by determination? Never ever take no for an answer … if I go to a corporation and the guy on the other side of the table says ‘no <name>, we’re not going to do that’, my mind immediately switches to ‘oh, why do you say that?’, because I’m then looking for feedback, I’m looking for them to give me all of the answers of what I should do to make that person change their mind, because they will, and they do, it’s just a matter of time.”

## 4. Discussion

When choosing animal welfare issues to pursue, animal welfare leaders overwhelmingly stated that above all, the issue must fit the strategic mandate and remit of the organization. By having a clear focus and organizational purpose, criteria can be developed that ensure proposed issues are in line with that mandate. Focusing on a few key issues core to the organization also ensures that resources (including expertise) are specific to the issue, and that attention and limited resources are not stretched too thin. While it may be tempting for animal welfare organizations to expand their focus to ever more issues, particularly for increasing donor pool and donor generosity, it may not be helpful to the causes being represented. In addition, successfully promoted victories in more narrow and focused fields may also bring about increased donor generosity. Strategically focusing on key areas in which an organization does well is a sentiment also reflected in product marketing strategy [15]. Likewise, business strategy often warns against growing beyond capacity and over diversification [16]. Second to this, many leaders stated a pragmatic and utilitarian-based approach of “best for the most”, also considering intensity of suffering, as an important criterion. In an industry that affects the lives of approximately 29 billion animals (chickens, ducks, cattle, pigs, sheep, turkey and buffalo) around the world at any given time [17], it is of little surprise that this often entailed factory farming programs to reduce the suffering of poultry, fish, and pigs. Identifying programs with the greatest impact to the most lives could be considered, in business sense, a good investment return. That is, for the same amount of investment, more lives could be positively impacted.

This is not to suggest that the animal welfare movement should turn away from other causes with fewer animals, such as captive wild animals, or companion animal welfare, as some species could serve as charismatic ambassadors for attitude change towards non-human animal species and could themselves bring about positive change. In some instances also, focusing on other species and issues may deliver more readily achieved positive outcomes. It is then suggested that core programs be chosen based on utilitarian criteria; however, opportunities to advocate for other causes when the socio-political environment allows should be considered. “Opportunity” was the third highest criteria that leaders used to decide if their organization would pursue an animal welfare issue. When considering cost benefit, if a positive outcome for the movement can be achieved, or an important government collaboration can be forged with reduced effort due to a presented opportunity, it is also recommended to be considered with high regard. In the absence of opportunities being directly presented to an organization, opportunities may be proactively identified by having intimate knowledge of the issues, and the socio-political environment in which the issue sits. In international animal welfare this also means a significant and well-founded insight into the country [18], culture and key stakeholders that surround the issue.

When identifying ideal leaders for the movement, a variety of traits were repeated by numerous leaders. Most notably, there was passion. While non-profit organizations vary when it comes to passion for their *charitable philosophy*, passion in their *purpose* may not be unique to non-profits. Some leadership researchers state that “human passion”, along with inspiration and vision of leaders directly drive corporate success [19]. Further to this, when investigating leadership styles in a post-recession environment, it has been found that passionate leaders were more successful in attracting funding, creating jobs, in particular within complex and challenging environments [20]. They suggest this leadership passion is attributable to project success being tied to the personal identity of the leader [20].

The “passion” referred to by leaders in this study was not as simple as just “passion for animal welfare”. Passion for animal welfare alone was not considered enough, and in fact, detrimental when not coupled with other key traits, such as focus and determination. Passion, as highlighted in this study, needs also to encompass a broad brand of passion that strives to see outcomes, supported by focus, drive, determination, and sense. As one leader states “just to wrap that in three words; passion, focus and determination”. 

Other than the ability to apply themselves in intimately understanding the issue and society in which their programs are focused, the third most common trait of an effective leader in animal welfare was the ability to build an engaged and competent team. This is echoed in leadership literature in other fields [10,19]. A repeating theme within this was the ability to lead the team from behind, utilizing evidence to move the cause forward and focusing on achieving outcomes instead of building up personal credentials. For this purpose, the traits make a full circle back to passion; passion for the cause, and passion for the outcomes … rather than passion for personal recognition. This should drive the selection of future leaders in the animal welfare movement. 

Non-profit leaders have decades of leadership literature and advice to draw upon from other fields; however, it is potentially underutilized. Inherent differences and challenges exist when leading non-profit organizations are compared to leading for-profit organizations; however, knowledge of basic human nature, psychology and cultural studies can be utilized across both. One leader stated, “I don’t really see it different from being a leader in any other … except you know, I think just by its nature we are people who love animals”. While this draws attention to the ability to learn “leadership lessons” from other fields, the unique differences and required skill mix, still suggests a need to better understand what makes successful leaders in international animal welfare. It is hoped that the findings of this study may assist to advise animal welfare organizations in the identification, recruitment and succession planning of ultimately successful leaders, along with strategic planning and identification of development opportunities within the movement. 

## 5. Conclusions 

Leaders most commonly identified animal welfare issues to pursue to success based on their fit with specific organizational remit and criteria. The purpose of this was to avoid stretching attention and resources too thin, to the point that each campaign loses efficacy. Additionally, it was recommended that animal welfare issues are also considered using a “best for the most” utilitarian framework, along with considering environmental and political opportunities that may arise (or are proactively discovered), to deliver positive outcomes for the movement. Passion, not only for the cause, was the number one trait identified with “good animal welfare leaders” within this study, and caution was issued against recruiting for “passion for the cause” alone. The passion for the cause must also be echoed with a passion for delivering outcomes and for engaging people. This passion must be backed by a focus, drive and determination to persist in the face of challenges and setback, to remain positive to people and the possibilities of collaboration. Creating an empowered team with the appropriate skills and knowledge and leading them from behind; instead of focusing on their own eminence in the field are also key, along with an ability and interest in intimately understanding and respecting the society in which the issue is presented. 

This study is an early start to better understanding the strategic ways of people and issues in the animal welfare movement, and further, more in-depth, research is recommended to allow the development of best practices within the industry. The aim of this study is to present findings that may be of use to the animal welfare movement, in a bid to assist the movement to become more effective in its strategy and operations. 

## Figures and Tables

**Table 1:** Summary and frequency of nodes (identified occurring theme).

Theme	Sources, out of 15	References within the Sources
Fit within organizational mandate	7	9
“Best for the most”	5	7
Opportunity for collaboration or leverage	4	8
Duration and intensity of suffering	4	5
Ability to contribute	4	4
Fit within targeted “focus”	3	4
“Biggest difference” and “widest impact”	3	3

**Table 2:** Themes and words cited in relation to the question “What makes a good international animal welfare leader?”.

Theme	Sources out of 15	References within the Sources	Frequent Word *	References
Passion	8	9	”Passion”	12
Understand the issue and society	7	9	”Understand”	17
Ability to build an engaged and competent team	6	8	”Team”	7
Bigger picture	5	7		
Collaborative	5	6	”Engage”	7
Flexible (reason and moderation)	4	5		
Focus	3	6	”Focus”	7
Communicator	3	6		
Driven	3	5	”Driven”	5
Involved	3	4	”Involved”	5
Compassion and empathy	3	4		
Persistence and determination	2	3		

* Words found within the top 100 most frequently used words leaders used in response to “what makes a good animal welfare leader?”.
